# Survey of New York City Resident Physicians on Cause-of-Death Reporting, 2010

**DOI:** 10.5888/pcd10.120288

**Published:** 2013-05-09

**Authors:** Barbara A. Wexelman, Edward Eden, Keith M. Rose

**Affiliations:** Author Affiliations: Barbara A. Wexelman, Edward Eden, St. Luke’s–Roosevelt Hospital Center, Columbia University College of Physicians and Surgeons, New York, New York.

## Abstract

**Introduction:**

Death certificates contain critical information for epidemiology, public health research, disease surveillance, and community health programs. In most teaching hospitals, resident physicians complete death certificates. The objective of this study was to examine the experiences and opinions of physician residents in New York City on the accuracy of the cause-of-death reporting system.

**Methods:**

In May and June 2010, we conducted an anonymous, Internet-based, 32-question survey of all internal medicine, emergency medicine, and general surgery residency programs (n = 70) in New York City. We analyzed data by type of residency and by resident experience in reporting deaths. We defined high-volume respondents as those who completed 11 or more death certificates in the last 3 years.

**Results:**

A total of 521 residents from 38 residency programs participated (program response rate, 54%). We identified 178 (34%) high-volume respondents. Only 33.3% of all respondents and 22.7% of high-volume residents believed that cause-of-death reporting is accurate. Of all respondents, 48.6% had knowingly reported an inaccurate cause of death; 58.4% of high-volume residents had done so. Of respondents who indicated they reported an inaccurate cause, 76.8% said the system would not accept the correct cause, 40.5% said admitting office personnel instructed them to “put something else,” and 30.7% said the medical examiner instructed them to do so; 64.6% cited cardiovascular disease as the most frequent diagnosis inaccurately reported.

**Conclusion:**

Most resident physicians believed the current cause-of-death reporting system is inaccurate, often knowingly documenting incorrect causes. The system should be improved to allow reporting of more causes, and residents should receive better training on completing death certificates.

## Introduction

The death certificate is a public document issued by a government body that declares the date, location, and cause of a person’s death (1). Local municipalities determine the form of the death certificate. Death certificates are important legal documents and public health tools. Death certificates are used by public health researchers for identification of the leading causes of death, for surveillance of disease patterns, and for identification of disease outbreaks (1,2). Death certificate data are also used to determine public health funding and clinical research priorities. Because the number of autopsies performed is decreasing, death certificates have become an even more important source of data on the causes of death of Americans (3–5).

Several researchers have studied patient medical charts for errors in cause-of-death reporting, and several have demonstrated inaccurate cause-of-death reporting among residents (3,6,7). One study found that only 56.9% of attending physicians, 56.0% of resident physicians, and 55.7% of medical students matched experts for the correct cause of death in clinical case studies, indicating a need for instruction at all levels of practice (3). Another study found that 45% of resident respondents incorrectly identified a cardiovascular event as the primary cause of death. This study also found that more experience in death certificate completion resulted in fewer errors in cardiac diagnosis (6). The Framingham Heart Study and other studies have indicated that coronary artery disease is overestimated on death certificates as a cause of death in the general population by 24% and by as much as 2 times more in older patients. The extent to which such overestimation affects the quality of death certificate information and national mortality statistics is not known (4,8,9,10).

In the United States, approximately one-third of all deaths occur in hospitals (11). The death registration process involves physicians and hospital staff, funeral directors, the medical examiner’s office, and the health department bureau of vital statistics. The physician’s role in this process is to describe the chain of medical events or conditions leading to death and to certify the underlying cause of death. Hospital administrative staff helps to prepare the certificate and ensures it is completed in a timely manner. In most teaching hospitals, resident physicians are responsible for the completion of death certificates (12). The objective of this study was to examine the experiences and opinions of New York City physician residents on the accuracy of completing death certificates. A secondary objective was to identify sources of inaccuracy, limitations of the current system, and potential areas of improvement.

## Methods

All hospitals in New York City use the same cause-of-death reporting system. Death certificates are typically processed by personnel in the hospital admitting department. The admitting personnel may help physicians enter the data into the electronic death reporting system and advise physicians on rules for reporting cause of death. Certificates are then submitted to the New York City Department of Health and Mental Hygiene. In 2011, of 4,145 residents, 71% were in internal medicine, 14% in emergency medicine, and 15% in general surgery.

### Study design and sample

We administered an anonymous, Internet-based survey through SurveyMonkey (SurveyMonkey, Palo Alto, California) to resident physicians in New York City. We sent an electronic link to the survey by e-mail to all 70 internal medicine, emergency medicine, and general surgery residency program directors in the city. We surveyed both categorical and preliminary residents. A categorical residency is one in which training is somewhat diversified, but most training takes place in 1 specialty. Preliminary residents are first-year residents in a department (usually internal medicine or general surgery) who ultimately complete a residency in another more specialized field (eg, a first-year intern who completes a year in internal medicine and then enters a residency in anesthesia). Program directors were asked to forward the link for the electronic survey to residents in their program. The surveys were open for submission from May 15, 2010, through June 30, 2010. Residents were compensated for their participation with a $5 coffee gift card. This study protocol was approved by our institution’s institutional review board.

### Survey instrument

We developed a survey on residents’ overall experience in completing death certificates and their opinions of accuracy in cause-of-death reporting (Appendix). The survey also questioned residents about cause-of-death reporting for certain diseases and system-based issues. We included questions on experiences with hospital admitting departments and with the medical examiner’s office. Question types included a mix of yes-and-no responses, multiple choice, and free-text entry. Respondents were asked to estimate how many death certificates they had completed in the last 3 years, which allowed us to identify residents who had completed a higher number of death certificates than average. We designated residents as high-volume respondents if they reported completing 11 or more death certificates in the last 3 years. The survey was pilot-tested among residents at our institution.

### Data analysis

We defined a complete survey as one in which 80% of items were completed, in accordance with the Council of American Survey Research Organizations (13,14). We used χ^2^ and Wilcoxon tests to assess the association between respondent characteristics and their responses. We compared the responses of high-volume residents with the responses of all residents to identify trends among more experienced residents. We defined significance as *P* < .05; all tests were 2-sided. We conducted analyses by using Microsoft Excel version 2003 (Microsoft Corp, Redmond, Washington) and SAS/Stat software version 9.1 (SAS Institute Inc, Cary, North Carolina).

## Results

From 38 residency programs and 26 institutions in New York City, 521 residents responded to the survey; the response rate by program was 54%. Of participating programs, the mean resident response rate by program was 19% (range 2%–52%). One-third of respondents (34% [178]) were high-volume respondents.

Respondents were evenly distributed by postgraduate year and by sex (Table 1). Most respondents (91.7%) were categorical residents; 8.3% of respondents were preliminary residents. Most respondents were internal medicine residents (75.0%), followed by general surgery (12.5%) and emergency medicine residents (9.8%). This distribution of specialties is similar to the distribution found in residency programs in New York City.

**Table 1:** Characteristics of Survey Respondents (n = 521), by Residency Type, Study of New York City Resident Physicians on Cause-of-Death Reporting System, 2010^a^

Characteristic	Internal Medicine	Emergency Medicine	General Surgery	Other	All Respondents	*P* Value^b^
**Residents**	391 (75.0)	51 (9.8)	65 (12.5)	14 (2.7)	521	** —**
**Postgraduate year level**						
1	148 (37.9)	16 (31.4)	23 (35.4)	8 (57.1)	195 (37.4)	<.001
2	118 (30.2)	11 (21.6)	16 (24.6)	5 (35.7)	150 (28.8)	
3	116 (29.7)	20 (39.2)	8 (12.3)	0	144 (27.6)	
≥4	9 (2.3)	4 (7.8)	18 (27.7)	1 (7.1)	32 (6.1)	
**Type**						
Categorical	361 (92.3)	51 (100)	57 (87.7)	9 (64.3)	478 (91.7)	<.001
Preliminary	30 (7.7)	0	8 (12.3)	5 (35.7)	43 (8.3)	
**Sex**						
Male	192 (49.1)	20 (39.2)	36 (55.4)	8 (57.1)	256 (49.1)	.34
Female	199 (50.9)	31 (60.8)	29 (44.6)	6 (42.9)	265 (50.9)	
**Mean age (SD), y**	29.8 (3.2)	30.1 (3.4)	29.5 (2.9)	28.4 (3.2)	29.8 (3.1)	.22
**Hospital type**						
University	145 (37.1)	9 (17.6)	32 (49.2)	9 (64.3)	195 (37.4)	.009
University-affiliated	168 (43.0)	29 (56.9)	24 (36.9)	3 (21.4)	224 (43.0)	
Community	78 (19.9)	13 (25.5)	9 (13.8)	2 (14.3)	102 (19.6)	

a All values are no. (%) unless otherwise indicated.

b Wilcoxon and χ^2^ tests used to assess the association between respondent characteristics and their responses.

Only one-third of respondents (33.3%) believed the current system accurately documents correct cause of death (Table 2). Among the 3 major specialties surveyed, emergency medicine residents had the most negative opinion of the system’s accuracy: only 27.3% believed the system is accurate, compared with 32.9% of internal medicine respondents and 41.0% of general surgery respondents. Almost half of all respondents (48.6%) and 58.4% of high-volume respondents (Table 3) reported they had identified a cause of death on a death certificate that did not represent the true cause of death. More than half of the residents (54.0%) reported they were unable to list what they felt to be the correct cause of death after guidance from the admitting department in their hospital (Table 2). Only 20.8% of respondents were aware they could report “probable,” “presumed,” or “undetermined” as a cause of a death, and only 2.9% of respondents indicated they had ever updated a death certificate when new information (eg, microbiology culture results) became available. Only 39.8% of respondents reported receiving any training by their residency program in death certificate completion, and only 21.5% of respondents reported being directed to the New York City’s Board of Health mandatory training module on death certificate completion.

**Table 2:** Selected Survey Responses on Death Certificate Accuracy, by Residency Type, Study of New York City Resident Physicians on Cause-of-Death Reporting System, 2010^a^

Question	Internal Medicine (n = 391)	Emergency Medicine (n = 51)	General Surgery (n = 65)	Other (n = 14)	Total (n = 521)	*P* Value^b^
Have you ever written a diagnosis on a death certificate that you believed did not represent the true cause of death?	186/377 (49.3)	23/42 (54.8)	25/62 (40.3)	4/9 (44.4)	238/490 (48.6)	.65
In your medical opinion, in what percentage of cases would you estimate that the immediate cause of death that you wrote on the death certificate accurately represented why the patient died? mean % (no. of respondents)	70.4 (366)	63.4 (42)	67.9 (60)	83.1 (8)	69.9 (476)	.16
Have you ever treated a patient who in your medical opinion died of septic shock, where you were unable to use this as an accepted cause of death on the death certificate, and were thus forced to list an alternate cause of death?	263/366 (71.9)	31/42 (73.8)	34/60 (56.7)	5/8 (62.5)	333/476 (70.0)	.14
Have you ever treated a patient who in your medical opinion died of Acute Respiratory Distress Syndrome (ARDS), where you were unable to use this as an accepted cause of death on the death certificate, and were thus forced to list an alternate cause of death?	132/366 (36.1)	13/42 (31.0)	16/60 (26.7)	2/8 (25.0)	163/476 (34.2)	.46
In most hospitals the admitting department staff assists physicians in proper completion of death certificates. Have you ever felt you could not put the most accurate diagnosis on a death certificate because staff members said this diagnosis was not acceptable?	197/349 (56.4)	24/35 (68.6)	18/58 (31.0)	4/8 (50.0)	243/450 (54.0)	NC
Are you aware that in the event that the cause of death is not clear, you are allowed to use terms such as probable, presumed, or undetermined on a death certificate?	70/350 (20.0)	6/35 (17.1)	16/58 (27.6)	2/8 (25.0)	94/451 (20.8)	.70
Have you ever amended a death certificate with data/information later when it became available (ie, microbiology culture results or lab data)?	10/350 (2.9)	0/35	2/58 (3.4)	1/8 (12.5)	13/451 (2.9)	.29
Based on your overall experience, do you believe the current system of reporting cause of death on death certificates is accurate?	119/362 (32.9)	12/44 (27.3)	25/61 (41.0)	4/13 (30.8)	160/480 (33.3)	.31

Abbreviation: NC, not calculated.

a All values are numerator/denominator (%) of respondents who answered yes, except for the question of percentage of cases.

b Wilcoxon and χ^2^ tests used to assess the association between respondent characteristics and their responses.

**Table 3:** Selected Survey Responses of High-Volume^a^ Residents Compared With Responses of All Respondents, Study of New York City Resident Physicians on Cause-of-Death Reporting System, 2010

Question	All Respondents (n = 521)	High-Volume Respondents (n = 178)	*P* Value^b^
Numerator/denominator (%) of respondents who replied yes to, “Have you ever written a diagnosis on a death certificate that you believed did not represent the true cause of death?”	238/490 (48.6)	104/178 (58.4)	<.001
In your medical opinion, in what percentage of cases would you estimate that the immediate cause of death that you wrote on the death certificate accurately represented why the patient died? mean % (no. of respondents)	69.9 (476)	66.3 (176)	.009
Numerator/denominator (%) of respondents who replied yes to, “Have you ever treated a patient who in your medical opinion died of septic shock, where you were unable to use this as an accepted cause of death on the death certificate, and were thus forced to list an alternate cause of death?”	333/476 (70.0)	147/176 (83.5)	<.001
Numerator/denominator (%) of respondents who replied yes to, “Have you ever treated a patient who in your medical opinion died of Acute Respiratory Distress Syndrome (ARDS), where you were unable to use this as an accepted cause of death on the death certificate, and were thus forced to list an alternate cause of death?”	163/476 (34.2)	78/176 (44.3)	<.001
Numerator/denominator (%) of respondents who replied yes to, “Based on your overall experience, do you believe the current system of reporting cause of death on death certificates is accurate?”	160/480 (33.3)	39/172 (22.7)	<.001
**“If you selected no to the prior question, in what problem area do you feel there is the biggest room for improvement in the death certificate system?,” no. (%)**			
No. of respondents	349	140	NA
Physicians completing the certificates haphazardly	20 (5.7)	9 (6.4)	.07
Lack of education on how to fill in the certificates properly	91 (26.1)	30 (21.4)	
Medical examiner’s office	11 (3.2)	3 (2.1)	
Hospital/admitting department personnel	17 (4.9)	10 (7.1)	
The death certificate system not allowing certain diagnoses as causes of death	198 (56.7)	87 (62.1)	
Other	10 (2.9)	1 (0.7)	
**“If you ever listed a condition other than the one you believed was the most appropriate cause of death, what was the reason? Please mark ALL that apply.,” no. (%)** c			
No. of respondents	476	176	NA
This has never happened to me	88 (18.5)	20 (11.4)	NA
The system would not accept what I felt was the correct cause of death	298 (76.8)	132 (84.6)	
Personnel in the admitting office told me to put something else	157 (40.5)	77 (49.3)	
The medical examiner told me to put something else	119 (30.7)	54 (34.6)	
A senior resident or my attending told me to put something else	32 (8.2)	10 (6.4)	
I didn’t know the patient/it was on my “signout” from another team	79 (20.4)	37 (23.7)	
I did not know why the patient died/I took my best guess	95 (24.5)	40 (25.6)	
I just put something down that would be easily accepted	69 (17.8)	39 (25.0)	
Other	0	0	

Abbreviation: NA, not applicable.

a High-volume respondents were defined as residents who reported completing 11 or more death certificates in the past 3 years.

b Wilcoxon and χ^2^ tests used to assess the association between respondent characteristics and their responses.

c Percentages for responses to this question were based on the 388 “all” respondents and 156 high-volume respondents who did not choose “This has never happened to me.”

Of all respondents, 70.0% believed they were forced to identify an alternate cause of death when the patient died of septic shock (compared with 83.5% of high-volume respondents), and 34.2% believed they were forced to identify an alternate cause when the patient died of acute respiratory distress syndrome (compared with 44.3% of high-volume respondents). Overall, high-volume respondents had a more negative opinion of the accuracy of cause-of-death reporting than all respondents; whereas 33.3% of all respondents believed the system is accurate, only 22.7% of high-volume residents believed so (Table 3). Among respondents who reported identifying an alternate cause of death, 64.6% of respondents reported cardiovascular disease as the most frequent diagnosis assigned, 19.5% reported pneumonia, and 12.4% reported cancer. 

Most residents identified system-based issues as cause of failure. Of respondents who reported recording an inaccurate cause of death, 76.8% of all respondents and 84.6% of high-volume respondents believed that the death certificate system simply would not accept the true cause of death (Table 3). The next most common belief among those who reported identifying an alternate cause was that personnel in the hospital admitting department instructed them to do so; 40.5% all respondents and 49.3% of high-volume respondents believed this. Also among those reporting an alternate cause, 30.7% of all respondents and 34.6% of high-volume respondents indicated that the medical examiner instructed them to do so. Survey respondents identified transfer of care between residents as a problem; of respondents who reported identifying an alternate cause of death, 20.4% of respondents gave the reason, “I didn’t know the patient/it was on my ‘signout’ from another team,” and 17.8% of respondents reported, “I just put something down that would be easily accepted.”

The survey indicated several possible areas of improvement. Of respondents who did not believe the system was accurate, 56.7% of all respondents and 62.1% of high-volume respondents believed changing the system to allow certain conditions as acceptable causes of death is the problem area offering the “biggest room for improvement.” Respondents also suggested improving education: of respondents who did not believe the system was accurate, 26.1% cited lack of education on how to fill out death certificates properly.

## Discussion

This is the first comprehensive survey of medical, surgical, and emergency medicine residents on the current cause-of-death reporting system. It is also the first to document that physicians completing death certificates often knowingly complete them inaccurately. 

Most physicians responding to our survey perceived death certificates to be inaccurate. Physicians who completed 11 or more death certificates in the last 3 years (ie, physicians more experienced in cause-of-death reporting) had a more negative opinion of the accuracy of death certificates than physicians who completed 10 or fewer death certificates (ie, less experienced physicians). Residents reported being instructed to report causes of death they did not agree with by personnel (eg, hospital admitting personnel, medical examiner) not directly involved in the patient’s care. 

We identified several areas in the current system that may lead to inaccurate cause-of-death reporting. Consistent with results of prior studies (4,11,15–17), we found that residents overreported cardiac disease as a cause of death. Other causes of death, such as septic shock and acute respiratory distress syndrome, may be underrepresented. The cause-of-death reporting system in New York City does not recognize all symptoms or diagnoses as a cause of death. For example, a clinician cannot report septic shock as a cause of death unless the cause of septic shock (eg, *Escherichia coli* urinary tract infection) is identified. Our survey respondents recognized the limited number of diagnoses accepted by the system.

Previous studies explored interobserver variability in identifying cause of death on death certificates and attributed potential error to subjectivity of the medical practitioners. These studies concluded that better education was required to increase accuracy of death certificates (3,6,12,18). Although our study reinforces the idea that inaccuracy in cause-of-death reporting may arise from inadequate training of physicians, it also identifies systemic barriers that limit the ability of residents to report what they understand to be the most likely cause of death.

This study had several strengths. It was conducted in New York City, which is a large area and has many residency programs and hospitals that use the same cause-of-death reporting system. We were able to evaluate a large group of physicians completing death certificates within the same system. Because cause-of-death reporting systems are fairly similar throughout the United States, our findings may have broad relevance.

This study also had several limitations. Participation in our survey was voluntary, so several potential sources of bias exist. Although we sent the survey to every internal medicine, emergency medicine, and surgical residency program director in New York City, not all program directors may have forwarded the survey to their residents, and not all residents chose to respond. We could not confirm that all program directors forwarded the survey to their residents, but we were able to identify each program that responded. Residents who felt particularly negative or positive about death certificates may have had more or less interest in participating. Additionally, our survey was subject to recall bias; residents may have recalled only strongly positive or negative experiences, not accounting for the many times the system worked smoothly.

We have identified potential sources for improvement in the current cause-of-death reporting system. We suggest expanding the acceptable causes of death to all inpatient diagnoses codes and improving the training of resident physicians. We also recommend that residency programs review the way patient care is transferred between residents to ensure that the most effective signout processes (transfer of care at the beginning and end of a resident’s hospital shift) are used to limit death certificate errors.

Residents need better training in proper completion of death certificates, including cause-of-death identification, when and why causes should be amended, and the implications of cause-of-death data for their community. Historically, residents have not been well educated as to what they can and cannot put on death certificates, and most have not undergone formal training in death certificate completion (3). Although New York City has developed a mandatory online training module for physicians, only 21.5% of our survey respondents had completed the training module. We found no significant differences in responses between residents who completed training and those that did not. Forty percent of respondents reported receiving training through their residency program; respondents who received this training did not report the system as more accurate. 

Only one-third of the physician residents in our study believed the current cause-of-death reporting system in New York City is accurate. Residents routinely reported diagnoses on death certificates that did not match their medical judgments. These errors may have lasting effects on the public health priorities of the community. Reform is needed both in the training and education of residents and in the system itself. We hope these findings will contribute to improvements in the cause-of-death reporting system and eventually more appropriate distribution of health care dollars. 

## Appendix. Text of Survey Questionnaire

Dear House Staff Physician:

You are invited to complete a survey related to the way you complete death certificates in New York City. This is a short survey and should take only a few minutes to complete. Your answers are anonymous and all responses will be kept strictly confidential. There is no personal data on the survey to identify you. Your completed survey results will not be shared with anyone from your residency program. This survey is voluntary and you are not obligated to complete it if you do not wish to do so. Results of the survey will be made available to you in the future if desired. Thank you for your participation in this survey.

1. What type of residency program are you in?

- Internal medicine
- Emergency medicine
- Surgery
- Other ______________

2. What PGY level are you?

- PGY1
- PGY2
- PGY3
- PGY4 or higher

3. Are you currently a categorical or preliminary resident?

- Categorical
- Preliminary

4. How old are you?

- Age_____

5. Are you:

- Male
- Female

6. How would you describe your residency program?

- University hospital
- University-affiliated
- Community hospital

7. In your department, who is generally responsible for completing death certificates?

- Intern
- Resident
- Physician assistant or nurse practitioner
- Attending physician
- Other____________

8. In the last twelve months, approximately how many death certificates have you filled out? __________

9. In the last three years, approximately how many death certificates would you estimate you have filled out? _________

10. Approximately what percentage of patients for whom you filled out the death certificate were you the primary resident or intern caring for the patient during that hospitalization? _________ %

11. Approximately what percentage of patients for whom you filled out the death certificate were you on duty in the hospital at the time of their death? _________%

Now we want to know more about your experiences with the cause-of-death reporting of death certificates.

12. Have you ever written a diagnosis on a death certificate that you believed did not represent the true cause of death?

- Yes
- No

13. In your medical opinion, in what percentage of cases would you estimate that the immediate cause of death that you wrote on the death certificate accurately represented why the patient died? ________%

14. If you ever listed a condition other than the one you believed was the most appropriate cause of death, what was the reason? **Please mark ALL that apply.**

- This has never happened to me
- The system would not accept what I felt was the correct cause of death
- Personnel in the admitting office told me to put something else
- The medical examiner told me to put something else
- A senior resident or my attending told me to put something else
- I didn’t know the patient/it was on my “signout” from another team
- I did not know why the patient died/I took my best guess
- I just put something down that would be easily accepted
- Other ___________________________

Additional comments (optional): _______________________________________________________________________________________________________________________________________________________________________________________________________________

15. If you selected more than one reason for question 14, what was the most common reason?

- The system would not accept what I felt was the correct cause of death
- Personnel in the admitting office told me to put something else
- The medical examiner told me to put something else
- A senior resident or the attending told me to put something else
- I didn’t know the patient/it was on my “signout” from another team
- I did not know why the patient died/I took my best guess
- I just put something down that would be easily accepted
- Other_______________________________

16. Have you ever treated a patient who in your medical opinion died of septic shock, where you were unable to use this as an accepted cause of death on the death certificate, and were thus forced to list an alternate cause of death?

- Yes
- No

17. Have you ever treated a patient who in your medical opinion died of Acute Respiratory Distress Syndrome (ARDS), where you were unable to use this as an accepted cause of death on the death certificate, and were thus forced to list an alternate cause of death?

- Yes
- No

18. While completing death certificates, in the instances that you were unable to write what you felt was the most accurate immediate cause of death, and ultimately used a less appropriate diagnosis on the death certificates, what was the most frequent diagnosis you assigned?

- Cardiovascular disease (arteriosclerotic, hypertensive, or atherosclerotic)
- Pneumonia (bilateral, multilobar, or focal lobar)
- Cancer (whichever type)
- AIDS (either sexual risk factor or IV drug use–related)
- COPD or pulmonary emphysema
- Alzheimer’s dementia
- Chronic alcoholism
- Asthmatic bronchitis
- This has never happened
- Other __________________________________________
- Additional comments (optional): __________________________________________________________________________________________________________________________________________________

19. In the event that the immediate cause of death was unknown AND the medical examiner was notified, approximately what percentage of time did the medical examiner accept the case for autopsy? ____________%

20. If the immediate cause of death was unknown, what was the most common way you completed the immediate cause of death on the death certificate?

- Wrote diagnosis as “undetermined”
- Wrote in a diagnosis that would be easily accepted by the system, even though you did not believe this was what directly caused the patient’s death
- Wrote in a diagnosis of an underlying condition the patient had, even though you did not believe this was what directly caused the patient’s death
- Asked the medical examiner what to write
- Used a term such as probable or presumed with a likely cause of death
- Other _____________________________________________________
- Additional comments (optional): _________________________________________________________________________________________________________________________________________________________________________________________________________________________________

21. Has the medical examiner ever instructed you to put a certain diagnosis which you did not agree with as an immediate cause of death on a death certificate?

- Yes
- No

22. If the medical examiner instructed you to give a certain diagnosis as the immediate cause of death on a death certificate, what percentage of time did you **DISAGREE** with this recommendation as the cause of death? ________%

- Additional comments (optional): _________________________________________________________________________________________________________________________________________________________________________________________________________________________________

23. In a case where the medical examiner instructed you to write a diagnosis on the death certificate that you did not agree with, did you ever feel that they told you to write a specific diagnosis simply because it would be easily accepted by the system, even though it was not the most accurate cause of death?

- Yes
- No

24. In most hospitals the admitting department staff assists physicians in proper completion of death certificates. Have you ever felt you could not put the most accurate diagnosis on a death certificate because staff members said this diagnosis was not acceptable?

- Yes. In approximately how many instances did this occur? __________
- No
- Additional comments (optional): _________________________________________________________________________________________________________________________________________________________________________________________________________________________________

25. Are you aware that in the event that the cause of death is not clear, you are allowed to use terms such as probable, presumed, or undetermined on a death certificate?

- Yes
- No

26. Have you ever amended a death certificate with data/information later when it became available (ie, microbiology culture results or lab data)?

- No
- Yes, ________ (number of times)

27. What percentage of time do you believe the cause of death you wrote on the death certificate **WAS NOT** accurate? _________%

- Additional comments (optional): _________________________________________________________________________________________________________________________________________________________________________________________________________________________________

28. Have you undergone any training prior to January 1, 2010, on death certificate completion by your residency program or hospital?

- Yes
- No

29. Prior to January 1, 2010, have you ever been directed to the NYC Board of Health Module on death certificate completion?

- Yes
- No

30. Have you participated in the NYC electronic death certificate system using physician fingerprints?

- Yes
- No

31. Based on your overall experience, do you believe the current system of reporting cause of death on death certificates is accurate?

- Yes
- No

32. If you selected no to the prior question, in what problem area do you feel there is the biggest room for improvement in the death certificate system?

- Physicians completing the certificates haphazardly
- Lack of education on how to fill in the certificates properly
- Medical examiner’s office
- Hospital/admitting department personnel
- The death certificate system not allowing certain diagnoses as causes of death
- Other

33. Please fill out any additional thoughts or comments.

- ______________________________________________________________________________________________________________________________________________

This completes our survey.

Thank you.
